# Scoping ‘sex’ and ‘gender’ in rehabilitation: (mis)representations and effects

**DOI:** 10.1186/s12939-022-01787-1

**Published:** 2022-12-16

**Authors:** Jessica Ott, Sarah N. Champagne, Abdulgafoor M. Bachani, Rosemary Morgan

**Affiliations:** Johns Hopkins International Injury Research Unit, Health Systems Program, Department of International Health, Johns Hopkins Bloomberg School of Public Health, Baltimore, MD USA

**Keywords:** Sex, Gender, Rehabilitation, Health systems, Assistive technology, Intersectionality, Social science, Global health, Caregiving

## Abstract

**Background:**

Researchers have highlighted a large-scale global unmet need for rehabilitation. While sex and gender have been shown to interact with each other and with other social and structural factors to influence health and wellbeing, less is known about how sex and gender shape rehabilitation participation and outcomes within health systems.

**Methods:**

Using an intersectional approach, we examine literature that explores the relationship between sex and/or gender and rehabilitation access, use, adherence, outcomes, and caregiving. Following a comprehensive search, 65 documents met the inclusion criteria for this scoping review of published literature. Articles were coded for rehabilitation-related themes and categorized by type of rehabilitation, setting, and age of participants, to explore how existing literature aligned with documented global rehabilitation needs. Responding to a common conflation of sex and gender in the existing literature and a frequent misrepresentation of sex and gender as binary, the researchers also developed a schema to determine whether existing literature accurately represented sex and gender.

**Results:**

The literature generally described worse rehabilitation access, use, adherence, and outcomes and a higher caregiving burden for conditions with rehabilitation needs among women than men. It also highlighted the interacting effects of social and structural factors like socioeconomic status, racial or ethnic identity, lack of referral, and inadequate insurance on rehabilitation participation and outcomes. However, existing literature on gender and rehabilitation has focused disproportionately on a few types of rehabilitation among adults in high-income country contexts and does not correspond with global geographic or condition-based rehabilitation needs. Furthermore, no articles were determined to have provided an apt depiction of sex and gender.

**Conclusion:**

This review highlights a gap in global knowledge about the relationship between sex and/or gender and rehabilitation participation and outcomes within health systems. Future research should rely on social science and intersectional approaches to elucidate how gender and other social norms, roles, and structures influence a gender disparity in rehabilitation participation and outcomes. Health systems should prioritize person-centered, gender-responsive care, which involves delivering services that are responsive to the complex social norms, roles, and structures that intersect to shape gender inequitable rehabilitation participation and outcomes in diverse contexts.

**Supplementary Information:**

The online version contains supplementary material available at 10.1186/s12939-022-01787-1.

## Introduction

There is currently a large-scale global unmet need for rehabilitation. An analysis using the Global Burden of Disease (GBD) Study data from 2019 suggests that 2.41 billion individuals worldwide had conditions that would benefit from rehabilitation, which altogether contributed to 310 million years of life lived with disability (YLD), a 63% increase from 1990 due to population growth and aging [1]. While men and women had a similar prevalence of conditions that would benefit from rehabilitation—1.19 billion vs. 1.22 billion—women accounted for more YLDs (163 million) than men (146 million) [1]. This suggests that women could be more affected by a lack of access to and use of rehabilitation services. Because 77% of global physical rehabilitation needs are in low- and middle-income countries (LMICs) [2], where rehabilitation services are limited [3], a gender and/or sex disparity in global rehabilitation needs is likely to be pronounced in such settings.

Gender is multifaceted and includes concepts such as gender identity, gender expression, and social roles, norms, and expectations. Gender is enacted in everyday social practices that are embedded within social institutions and larger societies [4]. Gender identity is often understood in public health as a person’s deeply felt, internal, and individual experience of gender [5], although gender theorists like Connell [6] have importantly questioned whether “weld[ing] one’s personality into a united whole [may be] to refuse internal diversity and openness”. Gender expression is closely related to but not determined by gender identity and refers to how a person presents or expresses their gender. Sex typically refers to biological and bodily processes and characteristics like phenotype, genetic makeup, and hormone profile that can change throughout the life course due to medical procedures, environmental conditions, and events like menopause [7, 8]. It is important to acknowledge that while sex is related to biological and bodily processes and factors, it is also a construct that is used to categorize other people in our highly gendered society [9]. Diverse dimensions of sex and gender interact with each other and with other structural and social factors (such as age, disability, and race) to inform health and wellbeing [10, 11]. A large body of existing research has explored gender as a power relation and social determinant of health [10, 12], and how gender inequality is embedded within health systems [13–15], such as through inequitable: access to resources, roles and practices, norms and beliefs, decision-making power and autonomy, and laws, policies, and institutions. Less is known globally about the role of sex and gender (as both identity and power relation) and how they interact with each other and with other social structures and factors to shape rehabilitation access, use, adherence, and outcomes within health systems.

In this scoping review, we rely on the WHO’s definition of rehabilitation as “a set of interventions designed to optimize functioning and reduce disability in individuals with health conditions in interaction with their environment” [16]. Assistive technology (AT), which includes products that help to “maintain or improve an individual’s functioning and independence,” [17] and caregiving are important parts of rehabilitation that enable and promote social inclusion and participation and are accordingly encompassed in our definition of rehabilitation.

In this scoping review, we explore existing literature about the relationship between sex and/or gender and rehabilitation to understand what is known as well as to better understand gaps in the literature with the aim of informing the global rehabilitation and health systems research agenda. While we conceive of AT as an integral part of rehabilitation and hence not an independent aspect of care, the scoping review literature largely segregated AT from rehabilitation, which is reflected in the presentation of our findings. Furthermore, because conditions that would benefit from rehabilitation often require caregiving, and because women worldwide experience disproportionate expectations and adverse health effects related to providing care [18], we explored the gender dimensions of caregiving as they intersect with rehabilitation.

## Methods

This scoping review was developed following the methodological framework outlined by Arksey and O’Malley [19] and guided by the following research question: “How do sex and gender intersect with each other and with other structural and social factors to shape rehabilitation (inclusive of AT and caregiving) participation and outcomes?”

### Search strategy

Our scoping review employed a 15-year time span, responding to an influx of research and literature that emerged on gender and rehabilitation beginning in the mid-2000s. In order to capture as much of the relevant literature as possible, a broad search strategy inclusive of academic literature was employed. The following three databases were selected due to their perceived relevance to the research question: PubMed, Embase, and CINHAL. We did the initial search in PubMed and made minor modifications to adapt the search strategy to Embase and CINAHL, based on some combination of MeSH categories and other search terms within each database. MeSH categories and search terms included variants of the following gender and sex-related terms: ‘gender,’ ‘sex,’ ‘man,’ ‘woman,’ ‘boy,’ ‘girl,’ ‘male,’ ‘female,’ ‘intersex,’ ‘transgender,’ ‘gender equality,’ ‘gender equity,’ ‘gender identity,’ ‘gender role,’ ‘sexism,’ ‘sex disaggregated,’ ‘sex difference,’ ‘gender bias,’ ‘sex bias,’ ‘sex discrimination.’ Two authors determined the search strategies by reviewing search terms used in existing gender and health systems-related scoping reviews [20, 21] and with the support of two Johns Hopkins University Welch Medical Library informationists. The exact search terms are viewable in a supplemental file.

Each database search combined variants of the above sex and gender-related terms with variants of the following rehabilitation and assistive technology-related terms that were adapted to database-specific MeSH categories: ‘rehabilitation,’ ‘self-help device,’ ‘occupational therapy,’ ‘rehabilitation center,’ ‘physical and rehabilitation medicine,’ ‘psychiatric rehabilitation,’ ‘psychosocial rehabilitation,’ ‘rehabilitation nursing,’ ‘rehabilitation research,’ ‘telerehabilitation,’ ‘physical rehabilitation,’ ‘assistive device,’ and ‘assistive technology.’

Each database search included results in English and from both high-income countries (HICs) and LMICs, operating from an assumption that there would be limited results from LMICs but that an understanding of the scope of literature may help inform the global health research agenda related to gender and rehabilitation. We use the terms HIC and LMIC with the caveat that there is deep sociocultural heterogeneity between and within countries that fall beneath LMIC and HIC categories that limit their usefulness as comparative descriptors. The method yielded 4484 references across all databases.

### Study selection

**Fig. 1:**
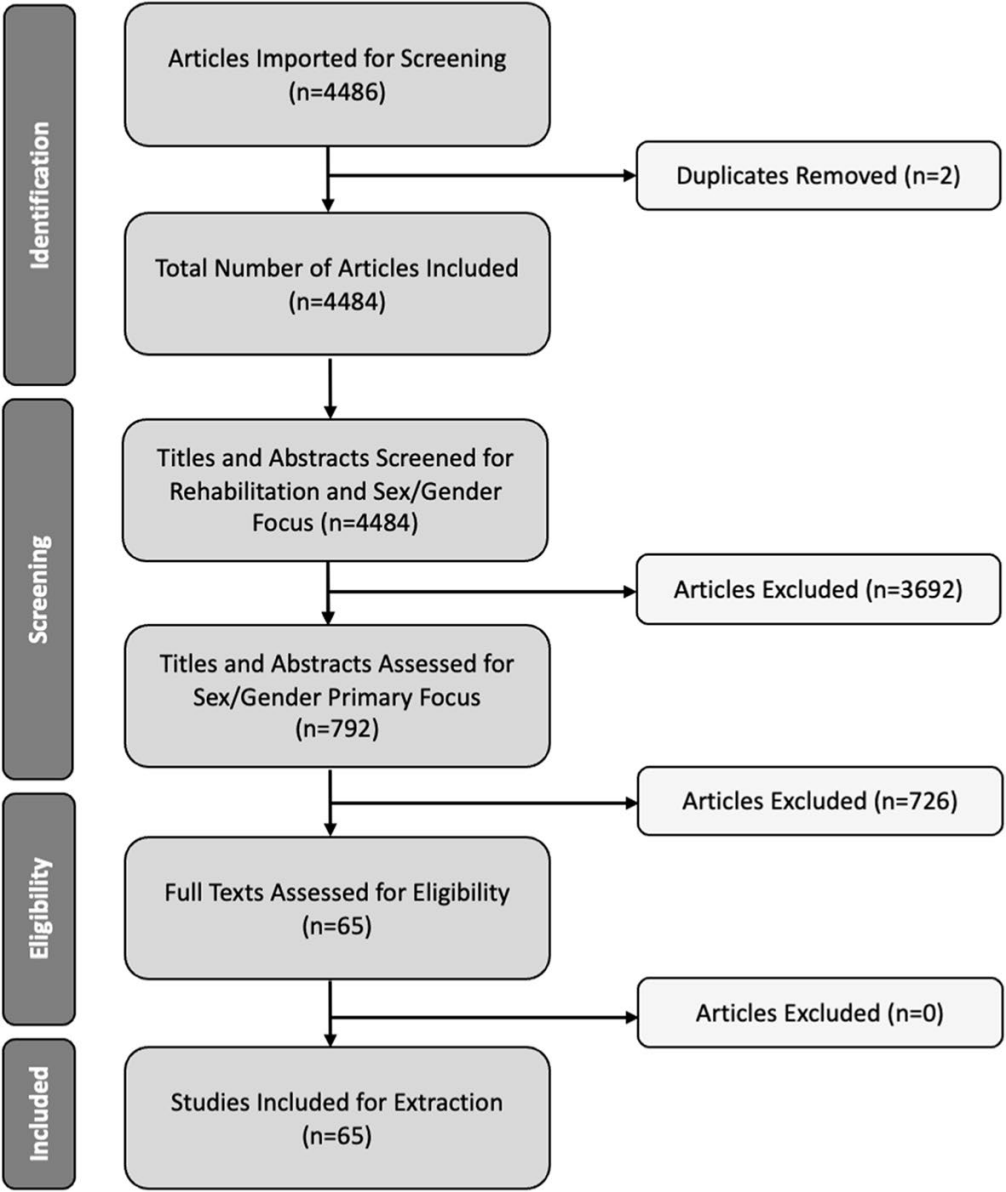
PRISMA flow diagram of the search and selection process

Because the search strategy yielded a large number of irrelevant references, a set of inclusion criteria was applied to all of the 4484 initial titles and abstracts. Two members of the team reviewed roughly 50 titles and abstracts collaboratively, to ensure consistency, before screening the remaining titles and abstracts separately. References were included after an initial title and abstract screening based on the following criteria: 1) focused on physical rehabilitation and/or post-rehabilitation functioning (including caregiver support and assistive technology access, use, satisfaction, and adherence), and 2) included a clear articulation of sex or gender differences or sex-disaggregated data related to rehabilitation participation and/or outcomes. The second criterion was included after it was found that many articles either controlled for sex/gender or reported sex/gender differences in passing, making it difficult to delineate any clear sex or gender differences. Beyond these inclusion criteria, the abstract and title reviewers intentionally chose not to focus on sex and gender differences in burdens of disease. While disease burden is tied to access, use, adherence, and outcomes, articles which focused on disease burden were not directly about rehabilitation, and as such, did not fit within the scope of this review. This inclusion and exclusion criteria yielded 792 studies from the original 4484 references. After engaging in a second round of reviewing titles and abstracts to determine whether articles included sex and/or gender as a major focus of the article, the reviewers were left with 65 references that underwent a full text review. After a full text review, all 65 references met the inclusion criteria, and rather than further refining our inclusion criteria to narrow down the final articles, we decided that it would be most fruitful to include all 65 articles that reached the step of full text screening in our review to ensure a more systematic exploration of current representations of sex and gender in rehabilitation-related literature. This method yielded 65 publications from the initial 4484 that underwent a full text review, among which all 65 were included in the scoping review (Fig. 1).

### Data extraction and synthesis

Two members of the study team participated in extracting and charting data from the final 65 publications. Study team members divided up the final 65 articles and scheduled frequent check-ins early on during the data extraction process to discuss how articles were being coded, refine the coding scheme, and work through discrepancies until concordance was reached. The following information was recorded from each publication: Author, title, year, journal, type of article, country, type of rehabilitation, methods, participants/target, number of participants/sample size, aims of study/paper, and whether or not the article adequately represented sex and gender based on the schema detailed in Table 2. Articles were also coded for the following rehabilitation-related themes: rehabilitation needs (RN), rehabilitation access and use (RAU), rehabilitation experiences (RE), rehabilitation adherence (RA), assistive technology (AT), health systems (HS), rehabilitation outcomes (RO), post-rehabilitation functioning (PRF), experiences of caregivers (EC), and other (O).

Articles were determined to adequately represent sex and gender if they did all of the following: (1) used the word gender to refer to gender-based social factors and/or sex to refer to sex-based biological factors; (2) presented sex and/or gender as nonbinary, which acknowledges the existence and experiences of people who fall outside of limited female/male and woman/man sex and gender categories; (3) defined the terms sex and/or gender, and (4) used gender-related terms like woman/man to refer to gender-related social factors and/or sex-related terms like female/male to refer to sex-based biological factors. The authors collaboratively developed the criteria for determining adequate representation of sex and gender. The two authors who were involved in data extraction reviewed articles after coding them to determine which criteria they did and did not fulfill. We began by reviewing a few articles together before reviewing the remaining articles independently. We also scheduled frequent check-ins early on in the testing process where we discussed questions that arose and further refined our criteria until we reached concordance. We included defining sex and/or gender due to widespread confusion and misrepresentations in public health literature about the difference between sex and gender, which has harmful implications for scientific understandings of the biological and social determinants of health. These criteria were loosely based on Heidari et al.’s [22] SAGER guidelines and recommendations, as well as on the charting categories of Williams et al.’s [21] scoping review on the integration of sex and gender considerations in health policy making. While the SAGER guidelines and Williams et al.’s (2021) charting categories provide a broad and helpful overview of sex and gender considerations for researchers, our schema (Table 2) provides a more detailed explanation of why certain guidelines are important and how to operationalize them. We recognize the limitations inherent in categorical understandings of sex and gender [4], the frequent inextricability of sex and gender [9, 23], and the necessity of exploring gender as intersectional [24] and relational [4], but sought to develop a preliminary framework/schema that would be implementable among public health researchers exploring sex and/or gender health disparities (Table 2).

In the presentation of results, this scoping review relied as much as possible on the sex and gender terms put forth by researchers, who often used the terms interchangeably and were unclear about their use of particular terms. We use the terminology sex/gender or sex and/or gender to reflect our inability to determine why authors were relying on particular terms and to reflect how they mutually affect and shape health and wellbeing [9, 23]. We do not intend our inclusion of studies or use of researchers’ terms to be seen as an endorsement of the common conflation of sex and gender or marginalization of sexual and gender minorities in public health research; however, without additional information beyond the publications, we were not able to sufficiently decipher authors’ misuse of sex and/or gender terms.

Where possible, we incorporated an intersectional approach into our review of existing literature. Our intersectional approach highlights authors’ mentions of associations between categorical quantitative variables as well as authors’ reflections on the compounding effects of social and structural forces on rehabilitation access, participation, adherence, and outcomes. In theory, an intersectional approach acknowledges that human experiences cannot be reduced to any one single factor or constellation of factors and that the intersectional effects of different social and structural factors on health are not additive but compounding [24]. In practice, however, we did not have the original data to be able to explore complex relations across identities and analytic categories and were therefore mostly limited to authors’ descriptions of associations between categorical quantitative variables. For example, while several literature reviews and articles described women who identify as a racial or ethnic minority as participating less than white women and men in rehabilitation [25–28], the articles generally did not describe how social factors like gender discrimination and racism might have mutually constituted each other to shape their rehabilitation participation, with the exception of a qualitative article that offered a richer explanation of how social and structural forces like gender discrimination, racism, and social class interact in a compounding way to shape access to pain rehabilitation [28]. This scoping review foregrounds sex and gender as analytic variables but includes other relevant social factors that intersect with sex and gender to inform rehabilitation participation and outcomes as they emerged in gender-focused literature.

## Results

### Overview

The scoping review yielded 65 sources (41 original research articles, nine literature reviews, and 15 miscellaneous references, including editorials and commentaries) that generally described worse rehabilitation access, use, adherence, and outcomes and a higher burden of caregiving among women compared to men (Table 1). However, existing literature has focused disproportionately on a few types of rehabilitation among adults in HIC contexts. For example, of the 65 total articles, 29 (45%) emphasized a sex and/or gender disparity in cardiac rehabilitation participation and outcomes, among which 27 were based in HICs. There is limited research on gender and/or sex differences in rehabilitation participation and outcomes for noncardiac conditions, in LMICs, and among children and adolescents. While 77% of global rehabilitation needs are based in LMICs [2], only seven articles (11%) were based in LMICs [27, 29–34]. An additional two (3%) global articles focused on both LMICs and HICs [35, 36], with a limited LMIC focus. The literature on assistive technology suggested differences in access and use between men and women depending on the device and country income. All of the articles that explored caregiving as it related to gender and rehabilitation—seven in total—focused on post-stroke caregiving in HICs (*n* = 7) [37–43]. Furthermore, only four articles (6%) explored rehabilitation participation and outcomes among children and adolescents [30, 31, 34, 44].

**Table 1:** Overview of scoping review literature

Article Characteristics	Frequency and Percentage N (%)	References
**Type of Rehabilitation/Article Emphasis**		
Musculoskeletal rehabilitation	3 (5)	[28, 45, 46]
Neurological (includes TBI, ABI, SCI, and stroke) rehabilitation	11 (17)	[23, 47–56]
Sensory (includes vision and hearing) rehabilitation	1 (1)	[57]
Cardiovascular rehabilitation	29 (45)	[25–27, 29, 35, 36, 58–80]
Broad rehabilitation	4 (6)	[30, 81–83]
Cancer rehabilitation	2 (3)	[84, 85]
Assistive technology	8 (12)	[31–34, 44, 86–88]
Caregiving (all post-stroke)	7 (11)	[37–43]
**Type of Article**		
Original research	38 (58)	[25, 27–34, 38–44, 46, 47, 49–52, 57, 60, 63, 66, 68, 69, 71–74, 77, 79, 84, 86–88]
Mixed methods	2 (3)	[33, 40]
Qualitative	9 (14)	[28, 29, 38, 46, 47, 60, 63, 71, 72]
Quantitative	27 (41)	[25, 27, 30–32, 34, 39, 41–44, 49–52, 57, 66, 68, 69, 73, 74, 77, 79, 84, 86–88]
Literature reviews	9 (14)	[35–37, 45, 61, 62, 64, 76, 78]
Other (i.e., Editorials, theoretical frameworks, commentaries, etc.)	18 (28)	[23, 26, 48, 53–56, 58, 59, 65, 67, 70, 75, 80–83, 85]
**Article Setting**		
Not Applicable	13 (20)	[23, 48, 53, 56, 58, 59, 70, 75, 80–83, 85]
Global (Mostly HICs, with limited LMIC representation)	2 (3)	[35, 36]
LMICs	7 (11)	[27, 29–34]
Sierra Leone	1	[34]
Iran	1	[27]
Indonesia	1	[29]
India	1	[33]
Ghana	1	[32]
Bangladesh	1	[31]
Multiple LMICs	1	[30]
HICs	45 (69)	[25, 26, 28, 37–47, 49–52, 54, 55, 57, 60–69, 71–74, 76–79, 84, 86–88]
United States	10	[25, 40, 42, 47, 49, 51, 65, 66, 77, 86]
United Kingdom	1	[43]
Taiwan	1	[88]
Sweden	5	[28, 38, 39, 41, 57]
Spain	1	[63]
Norway	2	[46, 52]
Italy	2	[50, 55]
Denmark	1	[84]
Canada	12	[44, 54, 60, 67–69, 71–74, 79, 87]
Multiple HICs	8	[26, 37, 45, 61, 62, 64, 76, 78]

Of the 65 total articles, no articles (*n* = 0) adequately represented sex and gender based on the schema created (Table 2). Fifty-five articles (85%) were consistent and accurate in their use ‘sex’ and/or ‘gender’ as an analytic category. However, only 14 articles (22%) used gender-related terms to refer to gender-related phenomena and sex-related terms to refer to sex-related phenomena. Only eight articles (12%) provided definitions for the commonly misunderstood terms ‘sex’ and/or ‘gender’ and a mere two articles (3%) presented sex and/or gender as nonbinary. As highlighted in Table 3., five articles (8%) met three out of the four criteria of the schema. Of those five articles, two (3%) did not meet the criteria “defined ‘sex’ and/or ‘gender,’” and three (5%) did not present sex and/or gender as nonbinary. All articles that inaccurately used the terms ‘sex’ and ‘gender’ interchangeably (*n* = 10) did not meet the remaining three criteria. Only one editorial [82] and one case report [47] acknowledged sexual (e.g., intersex) and gender minorities as sex and gender subcategories, which leads to a dearth of knowledge about how sex and gender interact with social and structural factors to shape rehabilitation participation and outcomes within health systems among sexual and gender minorities (Table 2). Our use of the term ‘sexual minorities’ here is not intended to refer to sexual orientation.

**Table 2:** Schema Determining adequate representation of sex and gender

Criteria	Explanation	Frequency & percentage of articles N (%)
Accurately used the terms ‘sex’ and/or ‘gender’	When authors use the word gender to refer to sex-based factors and/or sex to refer to gender-based factors, it contributes to an inadequate representation of sex and/or gender. ‘Sex’ typically refers to biological and bodily processes and characteristics like phenotype, genetic makeup, and hormone profile that can change throughout the life course due to medical procedures, environmental conditions, and events like menopause [7, 8]. It is important to acknowledge that while sex is related to biological and bodily processes and factors, sex is also a construct that is used to categorize other people in our highly gendered society [9]. ‘Gender’ is multifaceted and includes concepts such as gender identity, gender expression, and social roles, norms, and expectations. Gender is enacted in everyday social practices that are embedded within social institutions and larger societies [4]. To refer to gendered social characteristics as ‘sex’ may suggest that said characteristics are somehow inherent or natural. This has the potential to be problematic as it further entrenches social norms and suggests they are ‘natural’ or ‘right.’ For example, to refer to women’s social role as caregivers as a sex-based characteristic implies that women *should* and *must* continue to occupy those often un- or under-paid roles. Using sex/gender interchangeably similarly contributes to a false understanding of sex/gender as binary, which will be further unpacked immediately below.	**55 (85)**
Used the terms ‘sex’ and/or ‘gender’ in a nonbinary way	When authors present sex and/or gender as binary, usually in the form of categorical variables versus social constructs or processes, it obscures the existence and experiences of people who fall outside of the limited female/male and woman/man sex and gender categories and contributes to an inadequate representation of sex and/or gender. Sex as a construct is not limited to males and females, but also includes intersex people. Similarly, gender as an identity is not limited to women and men, but also includes people who are transgender, gender fluid, gender nonbinary, two spirit people, or one of many other genders that have existed across space and over time. While authors may be working with pre-existing data sets that do not include gender minorities or may not have been successful in recruiting gender minority participation in their studies, authors should still take care to acknowledge that sex and gender are not binary and to acknowledge the shortcomings of how sex and gender are framed in their data sets.	**2 (3)**
Defined ‘sex’ and/or ‘gender’	Sex and gender are frequently misunderstood terms. When they remain undefined in research, readers may be prone to confuse or conflate the terms. To avoid inadequate representation of sex/gender, authors should define what they mean when they use the terms sex/gender. Because sex and gender mutually affect and shape health and wellbeing, it is not always possible to disentangle the two and may be more appropriate at times to refer to sex/gender [9, 23]. In such instances, authors should clearly describe and justify their use of terms and be clear about the dimensions of sex and gender that are difficult to disentangle.	**8 (12)**
Accurately used gender- and/or sex-related terms	When authors use gender-related terms like woman/man to refer to sex-based factors and/or sex-related terms like female/male to refer to gender-related social factors, it contributes to an inadequate representation of sex and/or gender. Sex-related constructs like male/female/intersex generally refer to bodily and biological processes and factors, whereas gender-related constructs like man/woman/gender nonbinary person/transgender person/gender fluid person are generally used in reference to sociocultural identities.	**14 (22)**
Adequately represented sex and/or gender		**0 (0)**

**Table 3:** Frequencies and combinations of sex and gender representations

**Criteria for Adequate Sex and Gender Representation**	**Frequency and** **Percentage of** **Articles N (%)**
1. Accurately used the terms ‘sex’ and/or ‘gender’ AND 2. Used the terms ‘sex’ and/or ‘gender’ in a nonbinary way AND 3. Defined sex and/or gender AND 4. Accurately used gender- and/or sex-related terms	0 (0)
1. Accurately used the terms ‘sex’ and/or ‘gender’ AND 2. Used the terms ‘sex’ and/or ‘gender’ in a nonbinary way AND 3. Accurately used gender- and/or sex-related terms	2 (3)
1. Accurately used the terms ‘sex’ and/or ‘gender’ AND 2. Defined sex and/or gender AND 3. Accurately used gender- and/or sex-related terms	3 (5)
1. Accurately used the terms ‘sex’ and/or ‘gender’ AND 2. Defined sex and/or gender	5 (8)
1. Accurately used the terms ‘sex’ and/or ‘gender’ AND 2. Accurately used gender- and/or sex-related terms	9 (14)
1. Accurately used the terms ‘sex’ and/or ‘gender’	36 (55)
0. *Met none of the criteria*	10 (15)

### Rehabilitation access, use, and adherence

**Fig. 2:**
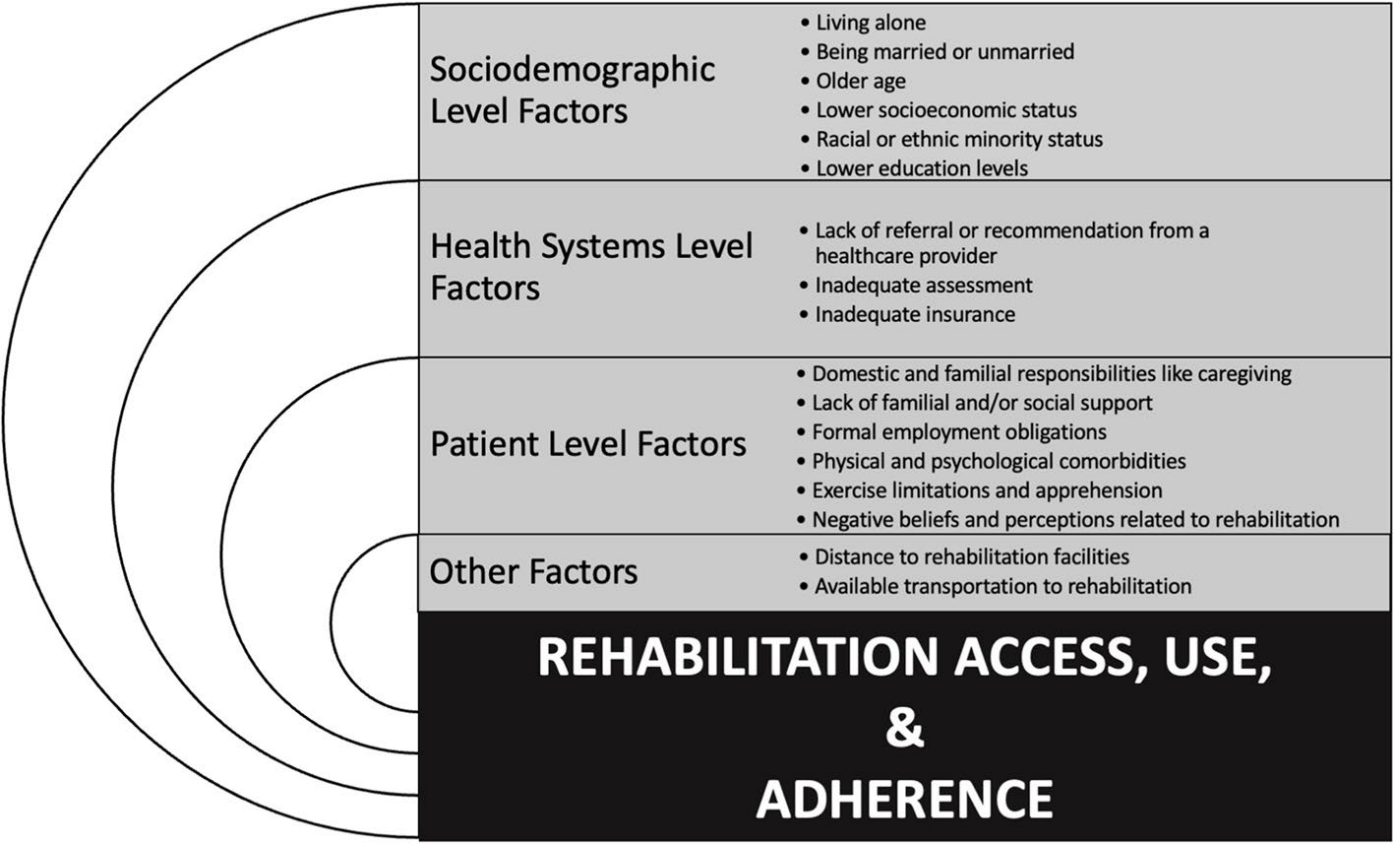
Factors influencing women’s rehabilitation access, use, and adherence

Our review revealed lower rates of rehabilitation participation among women than men, with the exception of women participating more than men in cancer rehabilitation in Denmark [84] and in audiological rehabilitation in Sweden [57]. Sociodemographic, health system, patient, and “other” factors influence women’s rehabilitation access, use, and adherence (Fig. 2). Unfortunately, scoping review articles reflect a widespread problem in health literature where researchers name gender but mostly speak about women, with men appearing mainly as the statistical norm or privileged category [4]. Sociodemographic-level factors that influence this disparity in rehabilitation participation between men and women include: lower education levels [25, 66, 68], living alone [57, 60, 62, 68], being married [26], being unmarried [61, 72], older age [25–28, 58, 59, 61, 63, 65, 72, 79, 83], lower socioeconomic status [27, 28, 45, 58, 61, 72], and racial or ethnic minority status [25–28]. Health systems factors like a lack of referral or recommendation from a healthcare provider [26, 27, 29, 35, 47, 58–62, 64, 65, 77–81], which is compounded by racial or ethnic minority status [61, 62, 78], socioeconomic status [26, 27, 58, 62, 65, 66, 78], or a combination of racial or ethnic minority and socioeconomic status [62, 65, 78]; inadequate assessment [28, 65]; and inadequate insurance [25, 27, 29, 40, 58, 64, 67, 78, 79] were shown to negatively impact women’s participation in rehabilitation. Just as a lack of referral or recommendation by a health care provider serves as a barrier to rehabilitation access, enthusiastic referral or recommendation serves as a facilitator to access [29, 62, 78]. Referral interventions that are either automatic or that require providers to systematically go through a list of criteria to reduce bias in referral decisions have been successful in reducing a disparity between men and women in access to cardiac rehabilitation [27, 36, 58, 60, 68, 79, 81]. Patient-level factors that adversely affect women’s rehabilitation access, use, and adherence include domestic and familial responsibilities like caregiving [25, 26, 45, 59–65, 67–70, 79]; a lack of familial and/or social support [26, 60]; formal employment obligations [29, 45, 60, 63–65, 70, 71]; physical and psychological comorbidities [25, 26, 60–65, 67, 68, 72, 77–79]; exercise limitations and apprehension [25, 26, 28, 59, 62–65, 67, 70, 77, 79]; and negative beliefs and perceptions related to rehabilitation [26, 64, 68, 70, 79].

We separated sociodemographic-level factors from the patient-level factors to emphasize that even when they are used to describe individuals, they are often the result of larger social and structural forces. While some studies reported patient-level factors like women’s domestic and caregiving responsibilities, a lack of social support, and sociodemographic-level factors such as older age as barriers to rehabilitation access, use, and adherence, other studies reported women’s domestic and caregiving responsibilities [29], the presence of social support [25, 29, 45, 61, 63, 64, 68, 71], and older age [68] as facilitators to their participation in rehabilitation. Other major factors affecting women’s rehabilitation access, use, and adherence, as compared to men, are distance and transportation [27, 29, 60–65, 67, 78, 79]. Health systems have tried to mitigate a disparity in cardiac rehabilitation participation, with women generally having lower access, use, and adherence than men, through women-only [27, 29, 59, 68–70, 72] and home-based and hybrid rehabilitation models [27, 59, 62, 67, 69], with mixed results.

### Rehabilitation outcomes (including psychosocial outcomes) and post-rehabilitation functioning

While few studies in the scoping review reported on gender and sex differences in functional improvements, among those that did, women tended to have less functional gain during rehabilitation than men and similar improvements in psychosocial measures. That is to say that even when women overcame barriers negatively impacting access, use, and adherence to rehabilitation and successfully completed their programs, women reportedly showed less physical, functional improvements post rehabilitation compared to men participants. Articles that reported women as having less overall functional gain during rehabilitation included studies on stroke rehabilitation in the United States [49]; cardiac rehabilitation in Canada [73]; stroke rehabilitation in Italy [50]; and rehabilitation for nontraumatic spinal cord injury in the United States [51]. Only one study, on audiological rehabilitation in Sweden, reported women as experiencing greater functional gain than men [57]. Although women as a whole experienced less functional gain than men during rehabilitation, there were subareas where women experienced greater functional outcomes than men. In a United States-based study on stroke rehabilitation, for example, women discharged with better functional scores than men in expression and social interaction, eating, and bladder control, while men discharged with higher scores in upper extremity dressing, movement from one surface to another, walking, and stairs than women [49]. In another United States-based study about degenerative spinal disease, women were more independent at discharge than men in self-care and mobility, perhaps because men entered rehabilitation with lower functioning after having waited longer than women to seek care due to widespread masculine social norms that hinder men’s care seeking [51].

Studies exploring gender and/or sex differences in improvements in psychosocial outcomes reported mixed results. A Canadian study reported no sex differences in improvements in anxiety or depression following cardiac rehabilitation [73]. Another Danish study reported men as having more emotional unmet needs than women following cancer rehabilitation [84], while a Canadian study reported women’s many unmet psychosocial needs following cardiac rehabilitation [74]. Several articles highlighted the potential of women-only programs to confer an advantage for psychosocial and other health outcomes compared to mixed-sex cardiac rehabilitation groups [25, 69]. A commentary suggested that existing psychosocial measures may not incorporate gender differences in men’s expressions and experiences of anxiety, depression, or other mental health issues, which limits researchers’ ability to understand men’s post-rehabilitation psychosocial outcomes or unmet psychosocial needs [23].

The scoping review yielded limited literature exploring sex and/or gender differences in post-rehabilitation functioning, which the International Classification of Functioning, Disability, and Health (ICF) model describes as “*all body functions, activities and participation”* [89]*.* This scoping review only yielded studies on differences between men and women in return to work rates following rehabilitation, revealing a gap in the literature on people’s post-rehabilitation functioning in other social contexts. One study reported higher return to work rates among women with acquired brain injury in Norway, perhaps because more men than women work in managerial positions that require greater cognitive and communication functioning [52]. Another study described Canadian women who returned to work more quickly than men after rehabilitation for musculoskeletal pain as overburdened by domestic and formal employment responsibilities [45]. The above examples suggest how inequitable gender norms, roles, and relations play an important role in social participation and physical functioning following rehabilitation.

### Assistive technology

The scoping review revealed gender differences in AT use, which are influenced by gender inequities and norms, with conflicting results in HICs and LMICs. In HIC-based articles, women used mobility devices [44, 86, 87] and vision devices [87] more than men, while men used hearing devices more than women [87, 88]. A Canadian study suggested that men’s lower mobility device usage may be due to the perceived stigma associated with using such devices [44]. The same study highlighted that while women use mobility devices more than men, they are more likely to report unmet needs for electric wheelchairs, walkers, lift devices, grab bars and other aids than men, which suggests that women’s higher usage may not reach their higher need for such devices, or that men may be less likely to report an unmet need for AT [44]. Another United States-based study similarly pointed out that while women use canes more than men, when analyses are adjusted for factors related to disability and health behavior, women are less likely than men to use canes [86].

Unlike in HICs, where women use most forms of AT more than men, men and boys in LMICs use AT more than girls and women [31–34]. Studies in Ghana [32] and Sierra Leone [34] reported a disparity between women and men in assistive device usage, with significantly lower usage rates among women than men, although population-level rates of assistive device usage were overall very low. The Sierra Leone-based study reported that women who used lower-limb prostheses and orthoses were less satisfied with their devices than men [34]. Researchers in an India-based study about mobile device-based assistive technology for vision impairment, like screen readers, found that while women with vision impairments paid more for mobile devices and had less access to them than men, cellular devices increased their participation in the public sphere [33].

### Caregiving

Women’s disproportionate global caregiving burden [18] seemed to hold true for rehabilitation, although the scoping review yielded only seven articles focusing on the gender dimensions of post-rehabilitation caregiving, all focusing on stroke patients and their caregivers in HICs [37–43]. Existing literature tended to focus less on how gender dynamics shape the experiences of caregivers and more on how sex and gender categories, which were often conflated, influence caregiving measures like quality of life and caregiver burden. Scoping review literature was heteronormative and revealed that women both provide more care to and receive less care from partners who are men, in part because women face more societal pressure to provide care, live longer than men, have adverse health events that require rehabilitation at an older age, and tend to be younger than their partners [38, 39, 41]. Assuming a caregiving role brings significant changes to the structure of daily life [37], including uncertainty, unpredictability, the assumption of new roles and tasks, and social isolation [38, 40], which can lead to caregivers experiencing a lost sense of self [40], depressive symptoms [42], and/or caregiving burden [43]. Scoping review literature highlighted that women stroke caregivers are more likely to provide care to partners with severe disabilities [41], experience more disruptions to their daily activities [37], and have a higher rate of depression than caregivers who are men [42], although men stroke caregivers also experience caregiver burden [40] and a lower quality of life due to their experiences as caregivers [43]. One study from the review suggested that caregiver training prior to patient discharge reduced caregiver burden [43]. None of the articles focused on the effects of receiving care for patient recovery trajectories.

## Discussion

Our scoping review highlighted sex and/or gender inequities in rehabilitation participation and outcomes; AT access, use, and user satisfaction; and caregiving. It also highlighted gaps and limitations in existing research, such as a relative overemphasis on a gender disparity in cardiac rehabilitation participation and outcomes in HICs; a limited understanding of how sex and gender shape rehabilitation participation and outcomes among children and adolescents and among sexual and gender minorities; the frequent misrepresentation of sex and gender and the treatment of sex and gender as binary; and limited research on the gender dimensions of post-rehabilitation caregiving or on sex, gender, and assistive technology. Our scoping review also revealed the need for further research on social norms, roles, and structures that influence a gender disparity in rehabilitation participation and outcomes, including how gender power relations shape the allocation of household resources, the division of household labor, and provider gender, referral patterns, and clinical interactions with patients in different contexts, just to name a few. For example, while researchers have described women’s caregiving and domestic responsibilities as a barrier to their participation in rehabilitation, the underlying gender power relations in different contexts that shape the division of household labor and influence gendered social expectations surrounding who engages in domestic and caregiving tasks are less explored in the context of rehabilitation. Furthermore, while researchers have identified lower referral rates to cardiac rehabilitation among women, provider presumptions and implicit biases concerning the desire and ability of different groups of women to attend cardiac rehabilitation, which shape referral patterns, are less known. Also related to gender power relations in clinical settings, patient preferences regarding the gender of their rehabilitation provider and the extent to which they shape rehabilitation attendance and experiences are unknown.

Our review found that the existing literature on sex and/or gender and rehabilitation does not correspond with geographic or condition-based global rehabilitation needs. For example, much of the literature that we reviewed focused on a sex and gender disparity in access to and use of cardiac rehabilitation in HICs, which does not correspond with women’s global condition-based rehabilitation needs. To illustrate, while the most prevalent conditions globally that would benefit from rehabilitation are musculoskeletal disorders (1.71 billion people), with men and women having a similar prevalence rate [1], only three articles in the scoping review focused on the relationship between sex, gender, and rehabilitation for musculoskeletal conditions. Conversely, nearly half of the articles (29/65) focused on the relationship between sex, gender and rehabilitation for cardiac conditions, for which far fewer men and women globally (37 million) would benefit from rehabilitation [1]. Furthermore, while the Western Pacific has the highest need for rehabilitation services, and the need for rehabilitation is a global one [1], existing literature on gender and rehabilitation is overwhelmingly based in North America and Europe.

Where geographic variation existed in the scoping review, there were often different or conflicting findings related to gender and rehabilitation participation and outcomes between HICs and LMICs. For example, HIC-based studies described women’s domestic and caregiving responsibilities as a barrier to their cardiac rehabilitation participation [25, 26, 45, 59–65, 67–70, 79], while an Indonesia-based study described women’s domestic and caregiving roles as a facilitator to their participation [29]. Additionally, while women and girls tended to use AT more than men in HICs [44, 86, 87], the opposite was true in LMICs [31–34]. The limited geographic and topical focus of literature on gender and rehabilitation and inconsistencies in the literature between LMICs and HICs point to a large gap in existing knowledge and the problems inherent in applying research findings from HICs to interventions in LMICs.

A common issue in existing research on rehabilitation is that it has often conflated or inadequately represented gender and sex [23, 81], which has led to confusion about how sex and gender interact with each other and with other social and structural factors to shape health and wellbeing, and has resulted in less rigorous, accurate, and valid scientific findings [11]. The conflation and misrepresentation of sex and gender additionally contributes to false understandings of sex and gender as binary, which both obscures the health experiences of transgender, gender nonbinary, and intersex individuals, and often leads to the erroneous conclusion that health disparities between men, women, and other genders are biological when the link is social or some combination of biological and social [90]. Furthermore, the literature—particularly on caregiving—tended to be heteronormative, which obfuscated the experiences of non-spousal caregivers as well as the care needs and experiences of lesbian, gay, transgender, queer (or questioning), intersex, two-spirit, plus (LGBTQIA2S+) individuals.

To provide an example from the scoping review of how the treatment of sex and gender as interchangeable can contribute to a misunderstanding of health disparities as solely biologically rooted, an Italian study on sex disparities in post-stroke rehabilitation outcomes reported significantly better functional recovery among men, which they suggested may be due in part to *sex differences* (authors' term) like men’s greater muscular strength and older women’s reduced physical activity [50]. Challenging the notion that men’s greater muscular strength and women’s reduced physical activity are biological sex-based factors, the biologist and feminist theorist Ann Fausto-Sterling [91] has importantly demonstrated that boys and girls are socialized to engage in different physical activities from a young age, which, together with biological factors, shapes their physical strength and activity as adults. Researchers’ attribution of women’s worse rehabilitation outcomes to biology obscures how their gendered socialization has also shaped their health and care. It also obscures the role of care—who receives it and who provides it—and its relationship with rehabilitation outcomes.

Rehabilitation researchers can address the problematic conflation and misrepresentation of sex and gender by undertaking sex- and gender-based analyses (SGBAs) [81], a procedure which has been operationalized into the Sex and Gender Equity in Research (SAGER) guidelines [22]. While sex and gender mutually affect and shape health and wellbeing, it is not always possible to disentangle the two and may be more appropriate at times to refer to sex/gender [9, 23], which reflects how “living bodies are dynamic systems that develop and change in response to their social and historical contexts” [9]. However, whenever authors rely on the term sex/gender, they should be clear about the dimensions of sex and gender that are difficult to disentangle rather than risk conflating the two as interchangeable. Furthermore, SGBAs should be combined with an intersectional approach, which acknowledges the compounding effects of various social and structural factors on health behaviors, opportunities, and outcomes [24].

The concept of embodiment—which refers to the relationship between bodily processes and social structures [6], including how gender as a multidimensional structure shapes health and illness [4]— enables a better understanding of how biological and social factors interact to shape health and wellbeing. However, the predominance of categorical thinking—or the frequent breakdown of complex social identities and dynamics into categorical quantitative variables—has limited an ability among researchers to conceptualize and measure gender as a multidimensional structure. Categorical thinking can be helpful in breaking down how social categories interact with each other to shape health disparities, but it is limited in its ability to understand the social dynamics at play that create disparate health outcomes between different groups [4]. Like Connell [4], we acknowledge the important role of categorical thinking as a “first approximation in understanding gender” and echo longstanding calls among sociologists and anthropologists to focus much more on how gender and other social and structural inequalities and dynamics become embodied [4, 6, 92].

This scoping review revealed that the incorporation of social science perspectives and methodologies can greatly increase our understanding of how the complex and multidimensional sociocultural construct of gender influences people’s rehabilitation experiences and outcomes within health systems. For example, a Norwegian study relied on qualitative interviews with ten men to explore men’s gendered expressions of pain, which adhered to dominant norms of masculinity and men’s desire to express suffering and vulnerability [46]. The researchers suggested that a sensitivity among health care providers to the role of gender in men’s expressions of chronic pain can improve their experiences in pain rehabilitation. Another Swedish study relied on qualitative interviews with five men and five women to explore access to pain rehabilitation from an intersectional gender perspective, which revealed how people’s negotiations with the healthcare system, based on various social factors and identities, including gender and class, may have shaped physician assessments of their need for pain rehabilitation [28]. This type of social science research is important, albeit limited, in clinical rehabilitation research and interventions, and could influence more careful, gender-responsive clinical studies and interventions among larger populations and in different sociocultural settings with diverse constructions of gender.

Intersectional social science approaches can inform person-centered, gender-responsive care and services that are sensitive to the complex social norms, roles, and structures that intersect to shape gender-inequitable rehabilitation participation and outcomes in diverse contexts. Person-centered care, sometimes referred to as “patient-centered” or “client-centered” care [93], seeks to attend to the individual needs, preferences, and circumstances of the person receiving care [94]. However, there is considerable variation in opinion on how to understand and address those needs, preferences, and circumstances, and hence translate person-centered care into practice [94]. Intersectionality is a theoretical framework that examines how social identities—for instance, race, gender, ethnicity, disability, sexual orientation, and socioeconomic status—impact the individual’s experience, reflecting larger, compounding socio-structural systems [95]. This scoping review advocates for person-centered care to be gender-responsive and highlights intersectionality’s close attention to the patient’s unique situation, which may enable providers to more accurately and better deliver rehabilitative care. It is important that as rehabilitation providers implement an intersectional and gender-responsive approach to care, they are constantly and consistently self-reflective about how their own implicit biases may be influencing their provision of rehabilitative care and that they are person-centered in their determination of treatment regimens for individual patients.

## Limitations

There are a few limitations to this scoping review. First, due to the time-intensive process of reading each document, only one researcher read and coded each article. We mitigated this limitation by holding frequent check-ins early on during the data extraction process in order to discuss how we were coding articles, refine the coding scheme, and work through any discrepancies until we reached concordance. Additionally, because the codes were descriptive, there was less interpretive variability between reviewers than if they had been analytic. However, articles would have been more thoroughly reviewed if two or more researchers had read and coded them. Second, our goal in conducting a scoping review was to get an overview of existing literature, which meant that we did not evaluate the final 65 articles for methodological rigor and quality. Third, we relied on public health databases and may have missed some social science literature that is not catalogued in the databases, although our database selection ensured the inclusion of most major social science and medicine-focused journals. Fourth, we decided not to include ‘physical therapy’ as a search term because it yielded a large volume of sports medicine literature that was not relevant for our purposes, but we did our best to ensure that rehabilitation-related physical therapy literature was captured with our other search terms. Fifth, we only included English language articles and therefore did not capture literature written in other languages. Last, because we relied on rehabilitation-related search terms, we certainly missed relevant, non-rehabilitation-related research focusing on issues like the gender dimensions of caregiving or how gender influences access to care that would have enriched our understanding of how gender influences rehabilitation participation and outcomes within health systems.

## Conclusions

There is a significant gap in the current body of literature that limits our understanding of how sex and gender shape rehabilitation. While researchers have documented a sex and/or gender disparity in access to and use of particular types of rehabilitation, there is limited knowledge about how sex and gender interact with each other and with other social norms, roles, and structures to shape rehabilitation access, use, adherence, and outcomes, particularly in LMIC contexts and for noncardiac conditions. Researchers should seek to alleviate this gap in knowledge, while health systems can begin to respond to sex and gender gaps in rehabilitation participation and outcomes by implementing interventions that have shown promise in reducing these disparities and by prioritizing care that is person-centered and gender-responsive. Within health systems, systematic referral, home-based, hybrid, and women-only rehabilitation programs, and caregiving interventions have all shown some promise in alleviating sex and gender disparities in rehabilitation participation, outcomes, and caregiving burden. Person-centered, gender-responsive care involves delivering services that are responsive to the complex social norms, roles, and structures that intersect to shape gender inequitable rehabilitation participation and outcomes among individuals in diverse contexts. Social science and intersectional approaches will be essential to understanding how sex and/or gender interact with other social and structural factors, such as disability, to shape rehabilitation participation and outcomes and to informing better health systems interventions in response to these sex and gender gaps.

## Supplementary Information

**Additional file 1.**

**Additional file 2.**

## Data Availability

The datasets used and/or analyzed during the current study are available from the corresponding author on reasonable request.

## Abbreviations

ABI: Acquired brain injury
AT: Assistive technology
CINHAL: Cumulative index to nursing and allied health literature
GBD: Global burden of disease study
HIC: High-income country
ICF: International classification of functioning, disability and health
LGBTQIA2S +: Lesbian, Gay, Bisexual, Transgender, Queer and/or Questioning, Intersex, Asexual, Two-Spirit, and countless affirmative ways in which people choose to self-identify
LMIC: Low- and middle-income country
SAGER: Sex and gender equity in research guidelines
SGBA: Sex and gender-based analysis
SCI: Spinal cord injury
TBI: Traumatic brain injury
YLD: Years of healthy life lost due to disability

## References

[1] Cieza A, Causey K, Kamenov K, Hanson SW, Chatterji S, Vos T. Global estimates of the need for rehabilitation based on the global burden of disease study 2019: a systematic analysis for the global burden of disease study 2019. Lancet. 2020;396(10267):2006–17. 10.1016/S0140-6736(20)32340-0. 10.1016/S0140-6736(20)32340-0 PMC7811204 33275908

[2] Jesus TS, Landry MD, Hoenig H. Global need for physical rehabilitation: systematic analysis from the global burden of disease study 2017. Int J Environ Res Public Health. 2019;16(6):980. 10.3390/ijerph16060980. 10.3390/ijerph16060980 PMC6466363 30893793

[3] Jesus TS, Hoenig H. Crossing the global quality chasm in health care: where does rehabilitation stand? Arch Phys Med Rehabil. 2019;100(11):2215–7. 10.1016/j.apmr.2019.07.001. 10.1016/j.apmr.2019.07.001 31653285

[4] Connell R. Gender, health and theory: conceptualizing the issue, in local and world perspective. Soc Sci Med. 2012;74(11):1675–83. 10.1016/j.socscimed.2011.06.006. 10.1016/j.socscimed.2011.06.006 21764489

[5] WHO Headquarters. Gender and health. [Internet]. 2021. Available from: https://www.who.int/news-room/questions-and-answers/item/gender-and-health.

[6] Connell R (2009). Gender in world perspective.

[7] Cicero EC, Reisner SL, Merwin EI, Humphreys JC, Silva SG. The health status of transgender and gender nonbinary adults in the United States. PLoS One. 2020;15(2):e0228765. 10.1371/journal.pone.0228765. 10.1371/journal.pone.0228765 PMC7034836 32084144

[8] Greaves L, Ritz S. Sex, gender and health: mapping the landscape of research and policy. Int J Environ Res Public Health. 2022;19(5):2563. 10.3390/ijerph19052563. 10.3390/ijerph19052563 PMC8909483 35270255

[9] Fausto-Sterling A (2012). Sex/gender: biology in a social world.

[10] Phillips SP. Defining and measuring gender: A social determinant of health whose time has come. Int J Equity Health. 2005;4:11. 10.1186/1475-9276-4-11. 10.1186/1475-9276-4-11 PMC1180842 16014164

[11] Johnson JL, Greaves L, Repta R. Better science with sex and gender: facilitating the use of a sex and gender-based analysis in health research. Int J Equity Health. 2009;8(14). 10.1186/1475-9276-8-14. 10.1186/1475-9276-8-14 PMC2689237 19419579

[12] Krieger N. Genders, sexes, and health: what are the connections - and why does it matter? Int J Epidemiol. 2003;32(4):652–7. 10.1093/ije/dyg156. 10.1093/ije/dyg156 12913047

[13] Morgan R, George A, Ssali S, Hawkins K, Molyneux S, Theobald S. How to do (or not to do)... Gender analysis in health systems research. Health Policy Plan. 2016;31(8):1069–78. 10.1093/heapol/czw037. 10.1093/heapol/czw037 PMC6616028 27117482

[14] Percival V, Richards E, MacLean T, Theobald S. Health systems and gender in post-conflict contexts: building back better? Conflict Health. 2014;8(19). 10.1186/1752-1505-8-19.

[15] WHO (2019). Delivered by Women, Led by Men: A Gender and Equity Analysis of the Global Health and Social Workforce.

[16] Rehabilitation. Available from: https://www.who.int/news-room/fact-sheets/detail/rehabilitation. [cited 9 Sep 2021]

[17] Assistive technology. Available from: https://www.who.int/health-topics/assistive-technology#tab=tab_1. [cited 9 Sep 2021]

[18] Berg JA, Woods NF. Global women's health: a spotlight on caregiving. Nurs Clin North Am. 2009;44(3):375-84. 10.1016/j.cnur.2009.06.003. 10.1016/j.cnur.2009.06.003 19683098

[19] Arksey H, O’Malley L. Scoping studies: Towards a methodological framework. Int J Soc Res Methodol Theory Pract. 2005;8(1):19–32. 10.1080/1364557032000119616.

[20] Alcalde-Rubio L, Hernández-Aguado I, Parker LA, Bueno-Vergara E, Chilet-Rosell E. Gender disparities in clinical practice: are there any solutions? Scoping review of interventions to overcome or reduce gender bias in clinical practice. Int J Equity Health. 2020;19(1):166. 10.1186/s12939-020-01283-4. 10.1186/s12939-020-01283-4 PMC7510055 32962719

[21] Williams A, Lyeo JS, Geffros S, Mouriopoulos A. The integration of sex and gender considerations in health policymaking: a scoping review. Int J Equity Health. 2021;20(69). 10.1186/s12939-021-01411-8. 10.1186/s12939-021-01411-8 PMC7923641 33653362

[22] Heidari S, Babor TF, De Castro P, Tort S, Curno M. Sex and gender equity in research: rationale for the SAGER guidelines and recommended use. Res Integr Peer Rev. 2016;1(2). 10.1186/s41073-016-0007-6. 10.1186/s41073-016-0007-6 PMC5793986 29451543

[23] Colantonio A. Sex, Gender, and Traumatic Brain Injury: A Commentary. Arch Phys Med Rehabil. 2016;97(2 Suppl):S1–4. 10.1016/j.apmr.2015.12.002. 10.1016/j.apmr.2015.12.002 26804988

[24] Hankivsky O. Women’s health, men’s health, and gender and health: implications of intersectionality. Soc Sci Med. 2012;74(11):1712–20. 10.1016/j.socscimed.2011.11.029. 10.1016/j.socscimed.2011.11.029 22361090

[25] Beckie TM, Beckstead JW. The effects of a cardiac rehabilitation program tailored for women on their perceptions of health: a randomized clinical trial. J Cardiopulm Rehabil Prev. 2011;31(1):25–34. 10.1097/HCR.0b013e3181f68acc. 10.1097/HCR.0b013e3181f68acc PMC3018536 21037482

[26] Jackson L, Leclerc J, Erskine Y, Linden W. Getting the most out of cardiac rehabilitation: a review of referral and adherence predictors. Heart. 2005;91(1):10–4. 10.1136/hrt.2004.045559. 10.1136/hrt.2004.045559 PMC1768637 15604322

[27] Nalini M. Outpatient cardiac rehabilitation use after coronary bypass surgery in the west of Iran. J Cardiopulm Rehabil Prev. 2014;34(4):263–70. 10.1097/HCR.0000000000000070. 10.1097/HCR.0000000000000070 24977464

[28] Wiklund M, Fjellman-Wiklund A, Hammarström A, Stålnacke B-M, Lehti A. Access to rehabilitation: patient perceptions of inequalities in access to specialty pain rehabilitation from a gender and intersectional perspective. Glob Health Action. 2016;9(31542). 10.3402/gha.v9.31542. 10.3402/gha.v9.31542 PMC5002397 27569592

[29] Sutantri S, Cuthill F, Holloway A. “A bridge to normal”: a qualitative study of Indonesian women’s attendance in a phase two cardiac rehabilitation programme. Eur J Cardiovasc Nurs. 2019;18(8):744–52. 10.1177/1474515119864208. 10.1177/1474515119864208 31328533

[30] Barth CA, Wladis A, Blake C, Bhandarkar P, O’sullivan C (2020). Users of rehabilitation services in 14 countries and territories affected by conflict, 1988–2018. Bull World Health Organ.

[31] Borg J, Östergren P-O (2015). Users’ perspectives on the provision of assistive technologies in Bangladesh: awareness, providers, costs and barriers. Disabil Rehabil Assist Technol.

[32] Akuamoah-Boateng H (2013). Self-reported vision health status among older people in the Kassena-Nankana District, Ghana. Glob Health Action.

[33] Pal J, Lakshmanan M (2015). Mobile devices and weak ties: a study of vision impairments and workplace access in Bangalore. Disabil Rehabil Assist Technol.

[34] Magnusson L, Ramstrand N, Fransson EI, Ahlström G. Mobility and satisfaction with lower-limb prostheses and orthoses among users in Sierra Leone: a cross-sectional study. J Rehabil Med. 2014;46(5):438–46. 10.2340/16501977-1780. 10.2340/16501977-1780 24658314

[35] Oosenbrug E, Marinho RP, Zhang J, Marzolini S, Colella TJF, Pakosh M, et al. Sex Differences in Cardiac Rehabilitation Adherence: A Meta-analysis. Can J Cardiol. 2016;32(11):1316–24. 10.1016/j.cjca.2016.01.036. 10.1016/j.cjca.2016.01.036 27129618

[36] Samayoa L, Grace SL, Gravely S, Scott LB, Marzolini S, Colella TJF. Sex differences in cardiac rehabilitation enrollment: A meta-analysis. Can J Cardiol. 2014;30(7):793–800. 10.1016/j.cjca.2013.11.007. 10.1016/j.cjca.2013.11.007 24726052

[37] Green TL, King KM. The trajectory of minor stroke recovery for men and their female spousal caregivers: literature review. J Adv Nurs. 2007;58(6):517–31. 10.1111/j.1365-2648.2007.04321.x. 10.1111/j.1365-2648.2007.04321.x 17542802

[38] Gosman-Hedström G, Dahlin-Ivanoff S. ‘Mastering an unpredictable everyday life after stroke’--older women’s experiences of caring and living with their partners. Scand J Caring Sci. 2012;26(3):587–97. 10.1111/j.1471-6712.2012.00975.x. 10.1111/j.1471-6712.2012.00975.x 22332755

[39] Gosman-Hedström G, Claesson L, Blomstrand C (2008). Consequences of severity at stroke onset for health-related quality of life (HRQL) and informal care: A 1-year follow-up in elderly stroke survivors. Arch Gerontol Geriatr.

[40] Pierce LL, Steiner V, Alamina F, Onyekelu D, Stevenson S. Male caregivers report problems in caring at home after spouses survive stroke. Home Healthc Now. 2019;37(1):23–32. 10.1097/NHH.0000000000000705. 10.1097/NHH.0000000000000705 30608464

[41] Appelros P, Nydevik I, Terént A (2006). Living setting and utilisation of ADL assistance one year after a stroke with special reference to gender differences. Disabil Rehabil.

[42] Epstein-Lubow GP, Beevers CG, Bishop DS, Miller IW. Family functioning is associated with depressive symptoms in caregivers of acute stroke survivors. Arch Phys Med Rehabil. 2009;90(6):947–55. 10.1016/j.apmr.2008.12.014. 10.1016/j.apmr.2008.12.014 19480870

[43] McCullagh E, Brigstocke G, Donaldson N, Kalra L. Determinants of caregiving burden and quality of life in caregivers of stroke patients. Stroke. 2005;36(10):2181–6. 10.1161/01.STR.0000181755.23914.53. 10.1161/01.STR.0000181755.23914.53 16151029

[44] Lindsay S, Tsybina I (2011). Predictors of unmet needs for communication and mobility assistive devices among youth with a disability: the role of socio-cultural factors. Disabil Rehabil Assist Technol.

[45] Côté D, Coutu M. A critical review of gender issues in understanding prolonged disability related to musculoskeletal pain: how are they relevant to rehabilitation? Disabil Rehabil. 2010;32(2):87–102. 10.3109/09638280903026572. 10.3109/09638280903026572 21495273

[46] Ahlsen B, Mengshoel AM, Solbrække KN. Troubled bodies - troubled men: A narrative analysis of men’s stories of chronic muscle pain. Disabil Rehabil. 2012;34(21):1765–73. 10.3109/09638288.2012.660601. 10.3109/09638288.2012.660601 22394105

[47] Brundage JA, Williams RD, Powell K, Raab J, Engler C, Rosin N, Sepahpanah F (2020). An interdisciplinary sexual health rehabilitation program for veterans with spinal cord injury: case reports. Sex Disabil.

[48] Aloisi AM, Berlincioni V, Torta R, Nappi RE, Tassorelli C, Barale F (2016). The role of gender, psycho-social factors and anthropological-cultural dimensions on pain in neurorehabilitation. Evidence and recommendations from the Italian consensus conference on pain in neurorehabilitation. Eur J Phys Rehabil Med.

[49] Hay CC, Graham JE, Pappadis MR, Sander AM, Hong I, Reistetter TA. The impact of One’s sex and social living situation on rehabilitation outcomes after a stroke. Am J Phys Med Rehabil. 2020;99(1):48–55. 10.1097/PHM.0000000000001276. 10.1097/PHM.0000000000001276 PMC6920562 31343498

[50] Paolucci S, Bragoni M, Coiro P, De Angelis D, Fusco FR, Morelli D, et al. Is sex a prognostic factor in stroke rehabilitation? A matched comparison. Stroke. 2006;37(12):2989–94. 10.1161/01.STR.0000248456.41647.3d. 10.1161/01.STR.0000248456.41647.3d 17082475

[51] Kay E, Deutsch A, Chen D, Semik P, Rowles D, Kay E, et al. Effects of gender on inpatient rehabilitation outcomes in the elderly with incomplete paraplegia from nontraumatic spinal cord injury. J Spinal Cord Med. 2010;33(4):379–86. 10.1080/10790268.2010.11689716. 10.1080/10790268.2010.11689716 PMC2964026 21061897

[52] Aas RW, Haveraaen LA, Brouwers EPM, Skarpaas LS. Who among patients with acquired brain injury returned to work after occupational rehabilitation? The rapid-return-to-work-cohort-study. Disabil Rehabil. 2018;40(21):2561–70. 10.1080/09638288.2017.1354234. 10.1080/09638288.2017.1354234 28724317

[53] Brauer J, Coates A, Schroeder A (2010). Addressing sexuality for women with an SCI. OT Pract.

[54] Thomas FP, Murphy C. Addressing disparities in the care of women with spinal cord injury: The Canadian perspective. J Spinal Cord Med. 2019;42(sup1):3. 10.1080/10790268.2019.1657749. 10.1080/10790268.2019.1657749 PMC6781254 31573449

[55] Tsuda K. Sex hormones and stroke rehabilitation in men and women. Stroke. 2007;38(6):e32. 10.1161/STROKEAHA.106.479105. 10.1161/STROKEAHA.106.479105 17463312

[56] Harris JE, Colantonio A, Bushnik T, Constantinidou F, Dawson D, Goldin-Lauretta Y, et al. Advancing the health and quality-of-life of girls and women after traumatic brain injury: Workshop summary and recommendations. Brain Inj. 2012;26(2):177–82. 10.3109/02699052.2011.635361. 10.3109/02699052.2011.635361 22360523

[57] Turunen-Taheri S, Carlsson P-I, Johnson A-C, Hellström S. Severe-to-profound hearing impairment: demographic data, gender differences and benefits of audiological rehabilitation. Disabil Rehabil. 2019;41(23):2766–74. 10.1080/09638288.2018.1477208. 10.1080/09638288.2018.1477208 29893149

[58] Lavie CJ, Bennett A, Arena R. Enhancing Cardiac Rehabilitation in Women. J Women's Health. 2017;26(8):817–9. 10.1089/jwh.2017.6476. 10.1089/jwh.2017.6476 28613967

[59] Kuehn BM. Women may benefit from cardiac rehabilitation programs tailored to their specific needs. Circulation. 2017;135(6):612–3. 10.1161/CIRCULATIONAHA.116.027064. 10.1161/CIRCULATIONAHA.116.027064 28153997

[60] Rolfe DE, Sutton EJ, Landry M, Sternberg L, Price JA. Women’s experiences accessing a women-centered cardiac rehabilitation program: a qualitative study. J Cardiovasc Nurs. 2010;25(4):332–41. 10.1097/JCN.0b013e3181c83f6b. 10.1097/JCN.0b013e3181c83f6b 20539167

[61] Galati A, Piccoli M, Tourkmani N, Sgorbini L, Rossetti A, Cugusi L, et al. Cardiac rehabilitation in women: state of the art and strategies to overcome the current barriers. J Cardiovasc Med (Hagerstown). 2018;19(12):689–97. 10.2459/JCM.0000000000000730. 10.2459/JCM.0000000000000730 30379752

[62] Parkosewich JA. Cardiac rehabilitation barriers and opportunities among women with cardiovascular disease. Cardiol Rev. 2008;16(1):36–52. 10.1097/CRD.0b013e31815aff8b. 10.1097/CRD.0b013e31815aff8b 18091401

[63] Resurrección DM, Motrico E, Rubio-Valera M, Mora-Pardo JA, Moreno-Peral P. Reasons for dropout from cardiac rehabilitation programs in women: A qualitative study. PLoS One. 2018;13(7):e0200636. 10.1371/journal.pone.0200636. 10.1371/journal.pone.0200636 PMC6047805 30011341

[64] Resurrección DM, Motrico E, Rigabert A, Rubio-Valera M, Conejo-Cerón S, Pastor L, et al. Barriers for nonparticipation and dropout of women in cardiac rehabilitation programs: A systematic review. J Women's Health (Larchmt). 2017;26(8):849–59. 10.1089/jwh.2016.6249. 10.1089/jwh.2016.6249 28388314

[65] Daniels KM, Arena R, Lavie CJ, Forman DE. Cardiac rehabilitation for women across the lifespan. Am J Med. 2012;125(9):937.e1–7. 10.1016/j.amjmed.2011.10.028. 10.1016/j.amjmed.2011.10.028 22748403

[66] Sanderson BK, Shewchuk RM, Bittner V. Cardiac rehabilitation and women: What keeps them away? J Cardiopulm Rehabil Prev. 2010;30(1):12–21. 10.1097/HCR.0b013e3181c85859. 10.1097/HCR.0b013e3181c85859 20068418

[67] Sedlak TL, Humphries KH. Cardiac rehabilitation adherence: another gender-treatment paradox. Can J Cardiol. 2016;32(11):1283–5. 10.1016/j.cjca.2015.12.032. 10.1016/j.cjca.2015.12.032 27129617

[68] Cossette S, Maheu-Cadotte MA, Mailhot T, Fontaine G, Dupuis J, Cournoyer A, et al. Sex-and gender-related factors associated with cardiac rehabilitation enrollment: a secondary analysis among systematically referred patients. J Cardiopulm Rehabil Prev. 2019;39(4):259–65. 10.1097/HCR.0000000000000364. 10.1097/HCR.0000000000000364 30252783

[69] Midence L, Arthur HM, Oh P, Stewart DE, Grace SL. Women’s health Behaviours and psychosocial well-being by cardiac rehabilitation program model: A randomized controlled trial. Can J Cardiol. 2016;32(8):956–62. 10.1016/j.cjca.2015.10.007. 10.1016/j.cjca.2015.10.007 26850727

[70] Way KL, Reed JL. Meeting the needs of women in cardiac rehabilitation. Circulation. 2019;139(10):1247–8. 10.1161/CIRCULATIONAHA.118.037754. 10.1161/CIRCULATIONAHA.118.037754 30865482

[71] Angus JE, Dale CM, Nielsen LS, Kramer-Kile M, Lapum J, Pritlove C, et al. Gender matters in cardiac rehabilitation and diabetes: using Bourdieu’s concepts. Soc Sci Med. 2018;200:44–51. 10.1016/j.socscimed.2018.01.003. 10.1016/j.socscimed.2018.01.003 29421471

[72] Sutton EJ, Rolfe DE, Landry M, Sternberg L, Price JAD. Cardiac rehabilitation and the therapeutic environment: the importance of physical, social, and symbolic safety for programme participation among women. J Adv Nurs. 2012;68(8):1834–46. 10.1111/j.1365-2648.2012.06041.x. 10.1111/j.1365-2648.2012.06041.x 22697385

[73] Terada T, Chirico D, Tulloch HE, Scott K, Pipe AL, Reed JL. Sex differences in psychosocial and cardiometabolic health among patients completing cardiac rehabilitation. Appl Physiol Nutr Metab. 2019;44(11):1237–45. 10.1139/apnm-2018-0876. 10.1139/apnm-2018-0876 30958974

[74] Hurley MC, Arthur HM, Chessex C, Oh P, Turk-Adawi K, Grace SL. Burden, screening, and treatment of depressive and anxious symptoms among women referred to cardiac rehabilitation: a prospective study. BMC Womens Health. 2017;17(11). 10.1186/s12905-017-0367-1. 10.1186/s12905-017-0367-1 PMC5297254 28173855

[75] Ghisi GLM, Chaves GSDS, Bennett A, Lavie CJ, Grace SL. The paucity of data addressing the effects of cardiac rehabilitation on mortality and morbidity in women. Can J Cardiol. 2018;34(4):502.e1–2. 10.1016/j.cjca.2017.11.001. 10.1016/j.cjca.2017.11.001 29289403

[76] Ghisi GLM, Chaves GSDS, Bennett A, Lavie CJ, Grace SL. The effects of cardiac rehabilitation on mortality and morbidity in women: a meta-analysis attempt. J Cardiopulm Rehabil Prev. 2019;39(1):39–42. 10.1097/HCR.0000000000000351. 10.1097/HCR.0000000000000351 30252785

[77] Gee MA, Viera AJ, Miller PF, Tolleson-Rinehart S. Functional capacity in men and women following cardiac rehabilitation. J Cardiopulm Rehabil Prev. 2014;34(4):255–62. 10.1097/HCR.0000000000000066. 10.1097/HCR.0000000000000066 24977463

[78] McCarthy MM, Vaughan Dickson V, Chyun D. Barriers to cardiac rehabilitation in women with cardiovascular disease: an integrative review. J Cardiovasc Nurs. 2011;26(5):E1–10. 10.1097/JCN.0b013e3181f877e9. 10.1097/JCN.0b013e3181f877e9 21107274

[79] Grace SL, Gravely-Witte S, Kayaniyil S, Brual J, Suskin N, Stewart DE. A multisite examination of sex differences in cardiac rehabilitation barriers by participation status. J Women's Health (Larchmt). 2009;18(2):209–16. 10.1089/jwh.2007.0753. 10.1089/jwh.2007.0753 PMC2927522 19183092

[80] Scott LB. A call for intervention research to overcome barriers to women’s enrollment in outpatient cardiac rehabilitation programs. J Women's Health. 2010;19(11):1951–3. 10.1089/jwh.2010.2416. 10.1089/jwh.2010.2416 20932132

[81] Quigley A, McArthur C, Parker R, Gahagan J. Sex cells: why we need sex- and gender-based analyses in rehabilitation research now. Ann Phys Rehabil Med. 2020;64:(6):101472. 10.1016/j.rehab.2020.101472. 10.1016/j.rehab.2020.101472 33333208

[82] Jette AM (2020). The importance of collecting data on sexual orientation and gender identity (SOGI) in rehabilitation research. Phys Ther.

[83] Scherer M, Dicowden M. Organizing future research and intervention efforts on the impact and effects of gender differences on disability and rehabilitation: the usefulness of the International Classification of Functioning, Disability and Health (ICF). Disabil Rehabil. 2008;30(3):161–5. 10.1080/09638280701532292. 10.1080/09638280701532292 17852261

[84] Holm LV, Hansen DG, Johansen C, Vedsted P, Larsen PV, Kragstrup J (2012). Participation in cancer rehabilitation and unmet needs: A population-based cohort study. Support Care Cancer.

[85] Silver JK. Rehabilitation in women with breast cancer. Phys Med Rehabil Clin North Am. 2007;18(3):521–537. 10.1016/j.pmr.2007.05.003. 10.1016/j.pmr.2007.05.003 17678765

[86] Peterson LJ, Meng H, Dobbs D, Hyer K. Gender differences in mobility device use among U.S. older adults. J Gerontol B Psychol Sci Soc Sci. 2017;72(5):827–35. 10.1093/geronb/gbw081. 10.1093/geronb/gbw081 27495837

[87] Ishigami Y, Jutai J, Kirkland S. Assistive device use among community-dwelling older adults: A profile of Canadians using hearing, vision, and mobility devices in the Canadian longitudinal study on aging. Can J Aging. 2020;40(1):1–16. 10.1017/S0714980819000692. 10.1017/S0714980819000692 32419685

[88] Chang N-C, Dai C-Y, Lin W-Y, Chien C-Y, Hsieh M-H, Ho K-Y. Perception of hearing impairment and the willingness to use hearing aids in an elderly population in southern Taiwan: A community-based study. Int J Audiol. 2016;55(9):491–8. 10.1080/14992027.2016.1182651. 10.1080/14992027.2016.1182651 27218891

[89] WHO (2002). Towards a Common Language for Functioning, Disability and Health ICF Towards a Common Language for Functioning, Disability and Health: ICF The International Classification of Functioning, Disability and Health.

[90] Ott J, Morgan R (2021). Why sex and gender matter in interventions and research for rehabilitation within health systems.

[91] Fausto-Sterling A. The bare bones of sex: part 1 - sex and gender. Signs: Journal of Women in Culture and Society. 2005;30(2):1491–527. 10.1086/424932.

[92] Rapp R. Gender, body, biomedicine: how some feminist concerns dragged reproduction to the Center of Social Theory. Med Anthropol Q. 2001;15(4):466–77. 10.1525/maq.2001.15.4.466. 10.1525/maq.2001.15.4.466 11794871

[93] Yun DW, Choi JS. Person-centered rehabilitation care and outcomes: a systematic literature review. Int J Nurs Stud. 2019;93:74–83. 10.1016/j.ijnurstu.2019.02.012. 10.1016/j.ijnurstu.2019.02.012 30870614

[94] Gibson BE, Terry G, Setchell J, Bright FAS, Cummins C, Kayes NM. The micro-politics of caring: tinkering with person-centered rehabilitation. Disabil Rehabil. 2020;42(11):1529–38. 10.1080/09638288.2019.1587793. 10.1080/09638288.2019.1587793 30978119

[95] Bowleg L. The problem with the phrase women and minorities: intersectionality-an important theoretical framework for public health. Am J Public Health. 2012;102(7):1267–73. 10.2105/AJPH.2012.300750. 10.2105/AJPH.2012.300750 PMC3477987 22594719

