# Supplementary material for: Scoping ‘sex’ and ‘gender’ in rehabilitation: (mis)representations and effects
Source: Int J Equity Health. 2022 Dec 16;21:179. doi: 10.1186/s12939-022-01787-1 (PMC9756604; doi:10.1186/s12939-022-01787-1)
Supplement: Supplementary file 1 — Additional file 1. [file 12939_2022_1787_MOESM1_ESM.docx]

**Gender and Rehabilitation Search Terms: Conducted on 1/25/21**

**PubMed**

**Concept 1: Rehabilitation**

“rehabilitation”[mesh:noexp] OR “self-help devices”[majr] OR

“hospitals, rehabilitation”[majr] OR “occupational therapy”[majr] OR “physical and

rehabilitation medicine”[majr] OR “psychiatric rehabilitation”[majr] OR “rehabilitation

nursing”[majr] OR “rehabilitation research”[majr] OR “telerehabilitation”[majr] OR

“telerehabilitation”[tiab] OR “tele rehabilitation”[tiab] OR “physical rehabilitation”[tiab] OR

“psychiatric rehabilitation”[tiab] OR “assistive”[tiab]

**Concept 2: Gender**

“gender equity”[mesh] OR “gender identity”[mesh] OR “gender

role”[mesh] OR “sexism”[mesh] OR “sexism”[tiab] OR “gender equal*”[tiab] OR “sex

disaggregate*”[tiab] OR “sex difference*”[tiab] OR “gender”[tiab] OR “gender bias”[tiab]

OR “sex bias”[tiab] OR “sex discrimination”[tiab] OR “transgender*”[tiab] OR

“intersex”[tiab] OR (“women”[tiab] AND “men”[tiab]) OR (“female*”[tiab] AND

“male*”[tiab]) OR “sex”[tiab] OR (“boy”[tiab] AND “girl”[tiab]) OR (“boys”[tiab] AND

“girls”[tiab]) OR (“man”[tiab] AND “woman”[tiab])

**Embase**

**Concept 1: Rehabilitation**

‘assistive technology’/exp/mj OR ‘self help device’/exp/mj OR ‘rehabilitation

center’/exp/mj OR ‘occupational therapy’/exp/mj OR ‘rehabilitation medicine’/exp OR

‘psychosocial rehabilitation’/exp/mj OR ‘rehabilitation nursing’/exp/mj OR ‘rehabilitation

research’/exp/mj OR ‘telerehabilitation’/exp/mj OR ‘telerehabilitation’:ab,ti OR ‘tele

rehabilitation’:ab,ti OR ‘physical rehabilitation’:ab,ti OR ‘psychiatric rehabilitation’:ab,ti

OR ‘assistive’:ab,ti OR ‘self help device’:ab,ti

**Concept 2: Gender**

‘gender identity'/exp OR 'sex role'/exp/mj OR 'sexism'/exp OR 'gender bias'/exp OR

'gender and sex'/exp/mj OR 'sex difference'/exp/mj OR 'gender equit*':ab,ti OR 'gender

equal*':ab,ti OR 'sexism':ab,ti OR 'sex disaggregate*':ab,ti OR 'sex difference*':ab,ti OR

'gender':ab,ti OR 'gender bias':ab,ti OR 'sex bias':ab,ti OR 'sex discrimination':ab,ti OR

'transgender':ab,ti OR 'intersex':ab,ti OR ('women':ab,ti AND 'men':ab,ti) OR

('female*':ab,ti AND 'male*':ab,ti) OR 'sex':ab,ti OR ('girl':ab,ti AND 'boy':ab,ti) OR

('girls':ab,ti AND 'boys':ab,ti) OR ('man':ab,ti AND 'woman':ab,ti)

**CINAHL**

**Concept 1: Rehabilitation**

(MM “rehabilitation”) OR (MM “assistive technology devices”) OR (MM “rehabilitation

centers”) OR (MM “occupational therapy”) OR (MM “rehabilitation, psychosocial”) OR

(MM “rehabilitation nursing”) OR (MM “research, rehabilitation”) OR (MM

“telerehabilitation”) OR (TX “psychiatric rehabilitation”) OR (TX “physical rehabilitation”)

OR (TX “telerehabilitation”) OR (TX “tele rehabilitation”) OR (TX “assistive”)

**Concept 2: Gender**

(MM “gender identity”) OR (MM “gender role”) OR (MM “sexism”) OR (TI “gender

equit*”) OR (TI “gender equal*”) OR (TI “sexism”) OR (TI “sex disaggregate*”) OR (TI

“sex difference”) OR (TI “gender”) OR (MM “gender bias”) OR (TI “sex bias”) OR (TI

“sex discrimination”) OR (TI “transgender”) OR (TI “intersex”) OR (AB “gender equit*”)

OR (AB “gender equal*”) OR (AB “sexism”) OR (AB “sex disaggregate*”) OR (AB “sex

difference”) OR (AB “gender”) OR (AB “sex bias”) OR (AB “sex discrimination”) OR (AB

“transgender”) OR (AB “intersex”) OR ((TI “women”) AND (TI “men”)) OR ((TI “female*”)

AND (TI “male*”)) OR (TI “sex”) OR ((TI “boy”) AND (TI “girl”)) OR ((TI “boys”) AND (TI

“girls”)) OR ((TI “man”) AND (TI “woman”)) OR ((AB “women”) AND (AB “men”)) OR

((AB “female*”) AND (AB “male*”)) OR (AB “sex”) OR ((AB “boy”) AND (AB “girl”)) OR

((AB “boys”) AND (AB “girls”)) OR ((AB “man”) AND (AB “woman”))
